# Overexpression of circular RNA hsa_circ_0008621 facilitates colorectal cancer progression and predicts poor prognosis

**DOI:** 10.1002/ags3.12793

**Published:** 2024-03-22

**Authors:** Xiaohu Zhou, Lei Wu, Chunyan Tian

**Affiliations:** ^1^ Department of General Surgery The Affiliated Xuzhou Municipal Hospital of Xuzhou Medical University, Xuzhou first People's Hospital Xuzhou Jiangsu China

**Keywords:** colorectal cancer, hsa_circ_0008621, miR‐532‐5p, prognosis, progression

## Abstract

**Aim:**

To evaluate the potential role of serum and tissue hsa_circ_0008621 as a prognostic biomarker for CRC patients. Focused on the functional role of hsa_circ_0008621 in colorectal cancer (CRC).

**Methods:**

Serum and tissue hsa_circ_0008621 expression were quantified by qRT‐PCR in 157 CRC patients, as well as 100 serums from healthy controls. Serum and tissue hsa_circ_0008621 expression was evaluated for their prognostic role in CRC patients using Kaplan–Meier curves and Multivariate Cox proportional hazards analysis. To further characterize the biological role of hsa_circ_0008621 expression in CRC, in vitro hsa_circ_0008621 inhibition was performed and the effects on cellular growth, migration, invasion, apoptosis, and glycolysis were explored. Next, the downstream molecules for hsa_circ_0008621 were predicted.

**Results:**

Hsa_circ_0008621 expression was significantly upregulated in CRC tissues and serums. Serum hsa_circ_0008621 levels were significantly up‐regulated in advanced‐staged samples. High serum hsa_circ_0008621 expression was associated with shorter overall survival and recurrence‐free survival in CRC patients. Multivariate Cox regression analysis identified a high level of serum hsa_circ_0008621 expression as an independent prognostic factor with respect to overall survival and recurrence‐free survival. Loss of function assays for hsa_circ_0008621 in vitro led to a significant decrease in cell proliferation, migration, invasion, and glycolysis, but an increase in cell apoptosis. Hsa_circ_0008621 can sponge miR‐532‐5p, which targets SLC16A3.

**Conclusion:**

High level of serum hsa_circ_0008621 is associated with poor survival in CRC and promotes CRC progression, suggesting it to be a promising non‐invasive prognostic biomarker and novel therapeutic target in CRC patients.

## Graphical abstract

Hsa_circ_0008621 was related to the CRC prognosis. MiR‐532‐3p was a target for hsa_circ_0008621.

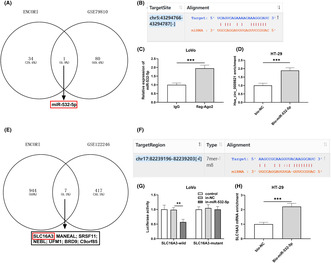

## 1 INTRODUCTION

Colorectal cancer (CRC) usually begins as a non‐cancerous growth (such as a polyp) on the inner lining of the intestine, whose cancerization affects the colon and the rectum.^1^ The estimated number of new cases for CRC is 153 020 in 2023 in USA.^2^ It kills 700 000 people annually, making it the third most deadly cancer in men and women.^3^ As a disease of modernity, its highest incidence rates are in developed countries.^4^ As the world becomes richer, colorectal cancer occurs throughout the world. As a heavily populated country, China undergoes rapid economic development, and so does the incidence of CRC.^5^ Moreover, the incidence of CRC looks set to increase in patients younger than age 50 for two decades.^6^ Young individuals are more often diagnosed at an advanced stage compared with their older counterparts.^6^ The 5‐year relative survival rate for CRC has increased to 65% from 2012 through 2018 across all stages.^2^ However, among people diagnosed with metastatic CRC, fewer than 20% of patients survive beyond 5 years from diagnosis.^7^ Long‐term survivorship challenges and lack of effective treatment options for the late CRC stages are some of the unique issues in the management of CRC.

Evidence is mounting that genetic and epigenetic alterations in CRC drive cancer cell biology properties, as well as shaping the heterotypic interactions across cells.^8^, ^9^ Epigenetic modifications such as non‐coding RNAs (ncRNAs) have central roles in the pathogenesis of various cancers, including CRC.^10^ NcRNAs such as microRNAs (miRNAs) and circular RNAs (circRNAs) can act as epigenetic regulators, preventing protein expression, though without protein‐coding ability, and influencing cancer‐related pathways at the post‐transcriptional level.^11^, ^12^ Many roles of circRNA have been discovered in CRC, including proliferation, metastasis, and apoptosis.^13^ In addition to cell biology, circRNAs also have unique associations with tumor size, staging, and overall survival in CRC, possessing the potential to serve as prognostic biomarkers.^14^ As the competitive endogenous RNA (ceRNA) hypothesis positing, circRNAs with shared miRNA binding sites can affect specific miRNA activity through sequestration, thereby indirectly mediating the posttranscriptional regulation of miRNA‐targeting mRNAs.^15^ For instance, circEZH2 (hsa_circ_0006357) facilitates the proliferation and migration of CRC cells, aggravating CRC progression and serving as a promising prognostic biomarker for CRC patients, via miR‐133b/IGF2BP2 axis.^16^ Hsa_circ_0008621 (hsa_circHMGCS1_016) locates in chr5: 43 292 575–43 297 268. Hsa_circ_0008621 has been reported to aggravate glioma via miR‐338‐5p/IKBIP axis,^17^ and contribute to development and immune tolerance by sponging miR‐1236‐3p to regulate CD73/GAL‐8 expression in intrahepatic cholangiocarcinoma.^18^ Therefore, the role and impact of hsa_circ_0008621 in CRC is an area of interesting scientific study.

Here, we aimed to evaluate the prognostic value of serum and tissue hsa_circ_0008621 as potential prognostic markers for CRC. Then, the involvement of hsa_circ_0008621 in CRC progression and glycolysis was explored.

## 2 MATERIALS AND METHODS

### 2.1 Screen of differentially expressed circRNAs in CRC by GEO database

After retrieving in GEO database, GSE138589, GSE147597, and GSE197991 datasets were analyzed by the criteria of logFC absolute value >1, and *p* ≦ 0.01. Then, the shared circRNAs were obtained by VENN diagram.

### 2.2 Target prediction

The downstream miRNAs for the obtained differentially expressed circRNAs were predicted from ENCORI platform. The differentially expressed miRNAs were screened from GSE79810, by the criteria of logFC absolute value >1, and *p* ≦ 0.01. The circRNA‐targeting differentially expressed miRNAs were obtained using VENN diagram. miR‐532‐5p‐target mRNAs were predicted from ENCORI platform, while the differentially expressed mRNAs were screened from GSE122246. The miR‐532‐5p‐targeting differentially expressed mRNAs were obtained using VENN diagram.

### 2.3 Clinical specimens

After the exclusion of patients with any neoadjuvant treatment or with too few samples, the study finally consisted of 157 newly diagnosed CRC patients at The Affiliated Xuzhou Municipal Hospital of Xuzhou Medical University in China. CRC serum and tissue specimens, along with 157 matched paracancerous normal tissues, were collected. In addition, serum from 100 healthy controls was collected. Written informed consent was obtained from all patients and the study was approved by the institutional review boards of all participating institutions. Extensive patient information was gained from paper and electronic medical records. Follow‐up information concerning recurrence/vital status, date, and cause of death was obtained after 5 years. The study was approved by the ethical committees of our institution. All participants were noticed and signed the written informed consent.

### 2.4 Cell culture and transfection

All cell lines and cell culture were purchased from American Type Culture Collection (USA): DMEM: F12 Medium for FHC, McCoy's 5a Medium Modified for HCT 116 and HT‐29, Leibovitz's L‐15 Medium for SW1116 and SW620, and F‐12 K Medium for LoVo. Cells were maintained at 37°C/5% CO_2_.

A negative control (scrambled, nontargeting) siRNA (siScr), two specific siRNAs for hsa_circ_0008621 (siCirc0008621‐1 and siCirc0008621‐2), a negative inhibitor control (in‐NC) and miR‐532‐5p inhibitor were purchased from Shanghai GeneBio Co., Ltd. Transfection was conducted using siPORT™NeoFX™ (Invitrogen, USA), using 2.5 μL of siRNA (1 μM) or 1.5 μL of inhibitor (10 μM) in 24‐well plates, for 48 h.

### 2.5 Total RNA isolation from serum, tissues, and cells

The total serum RNA was extracted from CRC serum and healthy serum using Plasma/Serum RNA Purification Mini Kit (Norgen Biotek, Canada). Weighted tissues were homogenized in TRIzol reagent (Invitrogen, USA) until no visible chunks, to extract total RNA according to the reagent manufacturer's protocol. Cultured cells were smoothly suspended in TRIzol reagent for isolation of total RNA.

### 2.6 Real‐time reverse transcriptase quantitative PCR (qRT‐PCR)

cDNA synthesis of RNAs was performed using GoScript™ Reverse Transcription System (Promega, Netherlands) according to provided instructions. Generated cDNAs were stored at −20°C or directly analyzed by MESA GREEN® RT‐qPCR kit (Eurogentec, Seraing, Belgium) on a QuantStudioTM7 Flex instrument. Melting curves were assayed to confirm the specificity of the primers. GAPDH and U6 served as internal reference genes. The 2^−ΔΔCt^ method was used for expression analysis of hsa_circ_0008621, miR‐532‐5p, and SLC16A3 mRNA.

### 2.7 Cellular proliferation

To measure proliferative activity, Cell Counting kit‐8 (CCK‐8) (Dojin Laboratories, Japan) reagent was used. Optical density of cells was measured at hours 0, 24, 48, 72, and 96, 2 h post the addition of 10 μL CCK‐8 at a wavelength of 450 nm on a microplate reader (TECAN GENios, Switzerland).

### 2.8 Cell migration and invasion in Boyden Chamber

Cell migration was performed on 8‐μm‐pore Transwells (Corning, USA) for 24 h, while cell invasiveness used Matrigel‐coated Transwells, as previously described.^19^ Migratory or invasive ability was evaluated by counting the cells migrating to the lower surface of the filters in six randomly chosen fields.

### 2.9 Cell apoptosis

Annexin V and PI dual‐staining methods based on Annexin V‐FITC Apoptosis Detection Kit (BioVision, USA) were employed to detect the proportion of apoptotic cells. LoVo or HT‐29 cells were digested with trypsin (without EDTA) and collected. The pellet was resuspended and labeled by Annexin V‐FITC for 15 min. The samples were kept on ice, and the PI solution was added 5 min before the measurement on a FACS CytoFLEX S (Beckman Coulter, USA).

### 2.10 Measurement of glucose uptake, lactate release, and intracellular ATP synthesis

After different transfection with siScr or siCirc0008621, LoVo or HT‐29 cells were seeded (50 000 cells per well) in 12‐well plates, undergoing serum starvation for 24 h. Afterward, cells were cultured with 100 nmol/L insulin for 30 min. The levels of glucose uptake, lactate release, and intracellular ATP production were determined according to the instructions of the glucose uptake colorimetric assay kit (BioVision, USA), lactate assay kit (BioVision), and the CellTiter‐Glo Luminescent Cell Viability Assay kit (Promega, USA), respectively, using a GloMax® Discover (Promega, USA) microplate reader.

### 2.11 Biotin pull‐down

LoVo or HT‐29 cells were transfected and isolated by centrifugation for the cytoplasmic lysate. Streptavidin‐coated magnetic beads (Invitrogen, USA) were blocked for 2 h in lysis buffer and washed twice. The prepared beads were co‐cultured with cytoplasmic lysate for 4 h at 4°C, and washed five times with lysis buffer. The pull‐down RNA that bound to the beads was isolated using Trizol reagent (Invitrogen, USA). The level of hsa_circ_0008621 or SLC16A3 mRNA in the bio‐NC or bio‐miR‐532‐5p was quantified by qRT‐PCR. The enrichment ratios of the pull‐down RNA to the control‐normalized input levels were then calculated.

### 2.12 RNA immunoprecipitation (RIP) assay

RIP assay was introduced to assess the enrichment of miR‐532‐5p by hsa_circ_0008621. Cells were lysed with RIP lysis buffer, before incubation with buffer containing anti‐Ago2‐conjugated protein A/G magnetic beads (Millipore, USA) or normal mouse immunoglobulin G (IgG) as the negative control. Then, immunoprecipitated RNA extracted from whole‐cell lysates was further analyzed by qRT‐PCR.

### 2.13 Luciferase assay

LoVo or HT‐29 cells were co‐transfected with 50 nM miR‐532‐5p inhibitor or inhibitor negative control and 50 ng of psiCHECK2 (Promega, USA) vector containing the binding sites between SLC16A3 and miR‐532‐5p. After 48 h of transfection, luciferase activities were measured using the manufacturer's instruction about the Dual Luciferase Assay System (Promega, USA) on a BioTek SynergyHTX (USA).

### 2.14 Statistical analyses

The software used statistical analyses included GraphPad Prism 9 and IBM SPSS Statistics 22. Student's t test was used to analyze normally distributed data and Mann–Whitney test was used to analyze the non‐normal distributed data, after Kolmogorov–Smirnov normality test. Comparisons of various clinicopathological factors between patients with high or low levels of hsa_circ_0008621 were done by chi‐square analysis. Kaplan–Meier curves were generated for the two subgroups of “low” and “high” expression using the mean relative expression value of hsa_circ_0008621 as cut‐off. Multivariate Cox proportional hazards models were used to identify independent predictive factors for patient overall or recurrence‐free survival (RFS). *p* < 0.05 was defined as the significance level.

## 3 RESULTS

### 3.1 Hsa_circ_0008621 expression was significantly upregulated in CRC

**FIGURE 1:**
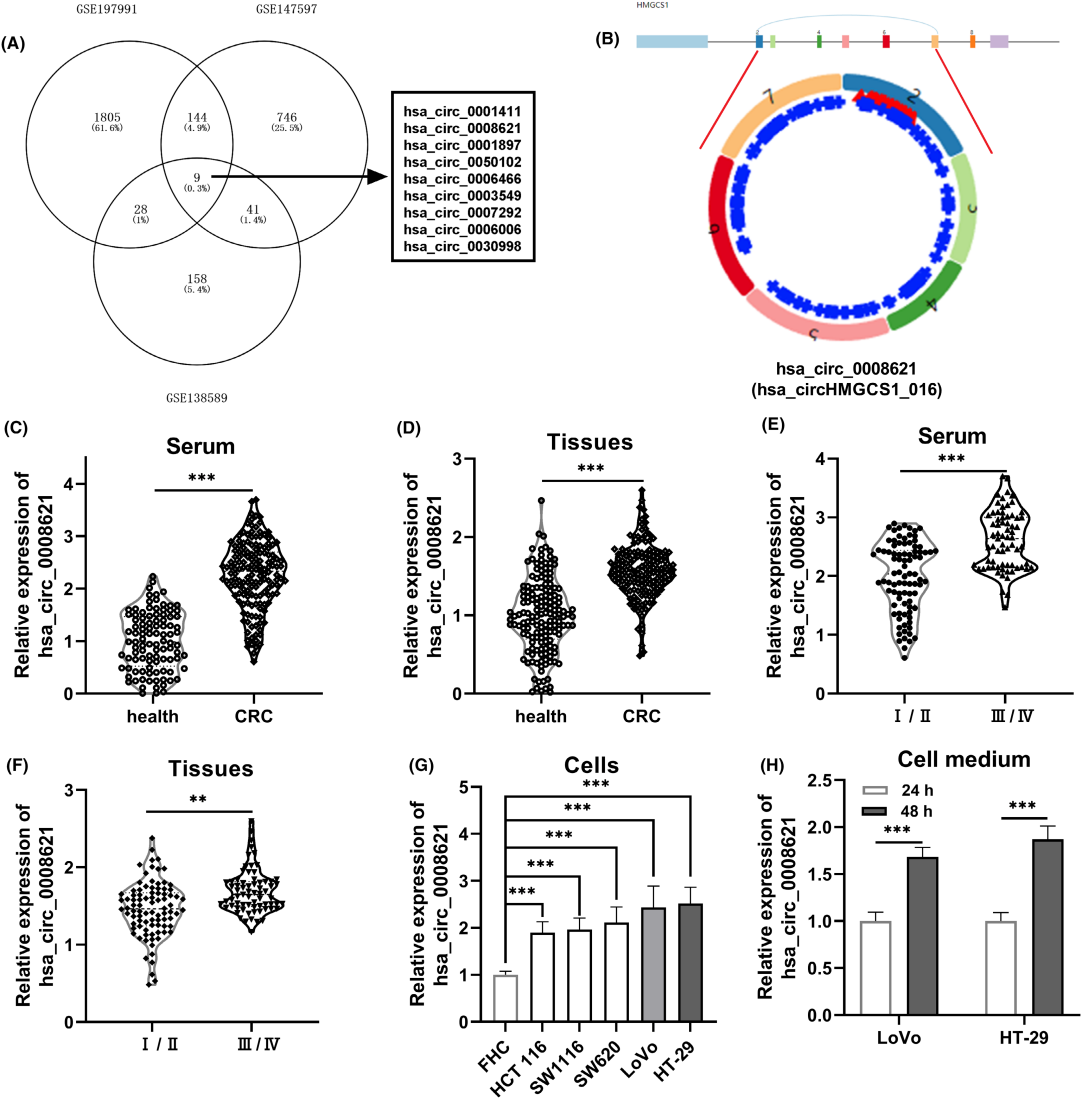
Hsa_circ_0008621 was upregulated in colorectal cancer (CRC). (A) Hsa_circ_0008621 was a shared circRNA among GSE197991, GSE147597, and GSE138589. (B) hsa_circ_0008621 originates from HMGCS1. (C) Expression status of hsa_circ_0008621 in serum from 157 CRC patients and 100 healthy controls. (D) Expression status of hsa_circ_0008621 in 157 paired tissues from CRC patients. (E) Expression status of hsa_circ_0008621 in serum at different CRC stages. (F) Expression status of hsa_circ_0008621 in tissues at different CRC stages. (G) Expression of hsa_circ_0008621 in CRC cell lines (HCT 116, SW1116, SW620, LoVo, and HT‐29) and normal human intestinal epithelial FHC cells. (H) The amount of hsa_circ_0008621 excreted in the LoVo and HT‐29 culture medium. ***p < 0.001, **p < 0.01.

There were nine differentially expressed circRNAs among GSE138589, GSE147597, and GSE197991 datasets, including hsa_circ_0001411, hsa_circ_0001897, hsa_circ_0050102, hsa_circ_0006466, hsa_circ_0003549, hsa_circ_0007292, hsa_circ_0006006, hsa_circ_0008621, and hsa_circ_0030998 (Figure 1A, Table S1). Hsa_circ_0008621 was transcribed by the host gene HMGCS1 on chromosome 5 (Figure 1B). To substantiate the finding from bioinformatics analysis, patients with different‐stage CRC were collected. All clinicopathological data of the 157 CRC patients, obtained from medical records, were summarized in Table 1. To investigate hsa_circ_0008621 expression in CRC tissues and serum, we performed qRT‐PCR analysis. There was a negatively correlation between hsa_circ_0008621 expression in CRC tissues and serum (*r* = 0.6988, *p* < 0.001; Figure S1). For comparison, the serum from healthy individuals and the adjacent normal tissues were also analyzed. As shown in Figure 1C,D, markedly higher levels of hsa_circ_0008621 were observed in CRC serum and tissues, compared to normal samples. Notably, in tissues and serum at advanced stages (III/IV), hsa_circ_0008621 expression was detected at higher levels compared with that at I/II stages (Figure 1E,F). The difference in expression in normal human intestinal epithelial FHC cells and different CRC cells was also observed (Figure 1G). Of the five CRC cell lines, LoVo and HT‐29 showed the highest expression level of hsa_circ_0008621. Further, LoVo and HT‐29 cell lines were cultured and hsa_circ_0008621 expression was investigated in the culture medium by qRT‐PCR, resulting in an increased level of hsa_circ_0008621 in the cell culture (Figure 1H).

**TABLE 1:** Correlations of tissue and serum hsa_circ_0008621 expression with clinicopathological characteristics of the colorectal cancer patients.

Characteristics	Patients (*n* = 157)	Serum hsa_circ_0008621		*p* value	Tissue hsa_circ_0008621		*p* value
		Low (*n* = 73)	High (*n* = 84)		Low (*n* = 79)	High (*n* = 78)	
Age							
≤65	77	38	39	0.482	41	36	0.472
>65	80	35	45		38	42	
Sex							
Female	68	27	41	0.136	31	37	0.300
Male	89	46	43		48	41	
Size							
≤Median	95	51	44	**0.025**	55	40	**0.019**
>Median	62	22	40		24	38	
T stage							
T1/T2	47	19	28	0.319	22	25	0.567
T3/T4	110	54	56		57	53	
N stage							
N0	87	47	40	**0.035**	48	39	0.175
N1/N2/N3	70	26	44		31	39	
M stage							
M0	125	64	61	**0.020**	68	57	**0.043**
M1	32	9	23		11	21	
TNM							
I/II	88	49	39	**0.009**	51	37	**0.031**
III/IV	69	24	45		28	41	
CEA							
≤5	48	20	28	0.421	20	28	0.150
>5	109	53	56		59	50	

*Note*: The bold texts indicated statistical significances.

### 3.2 High hsa_circ_0008621 expression was associated with poor prognosis in CRC patients

**FIGURE 2:**
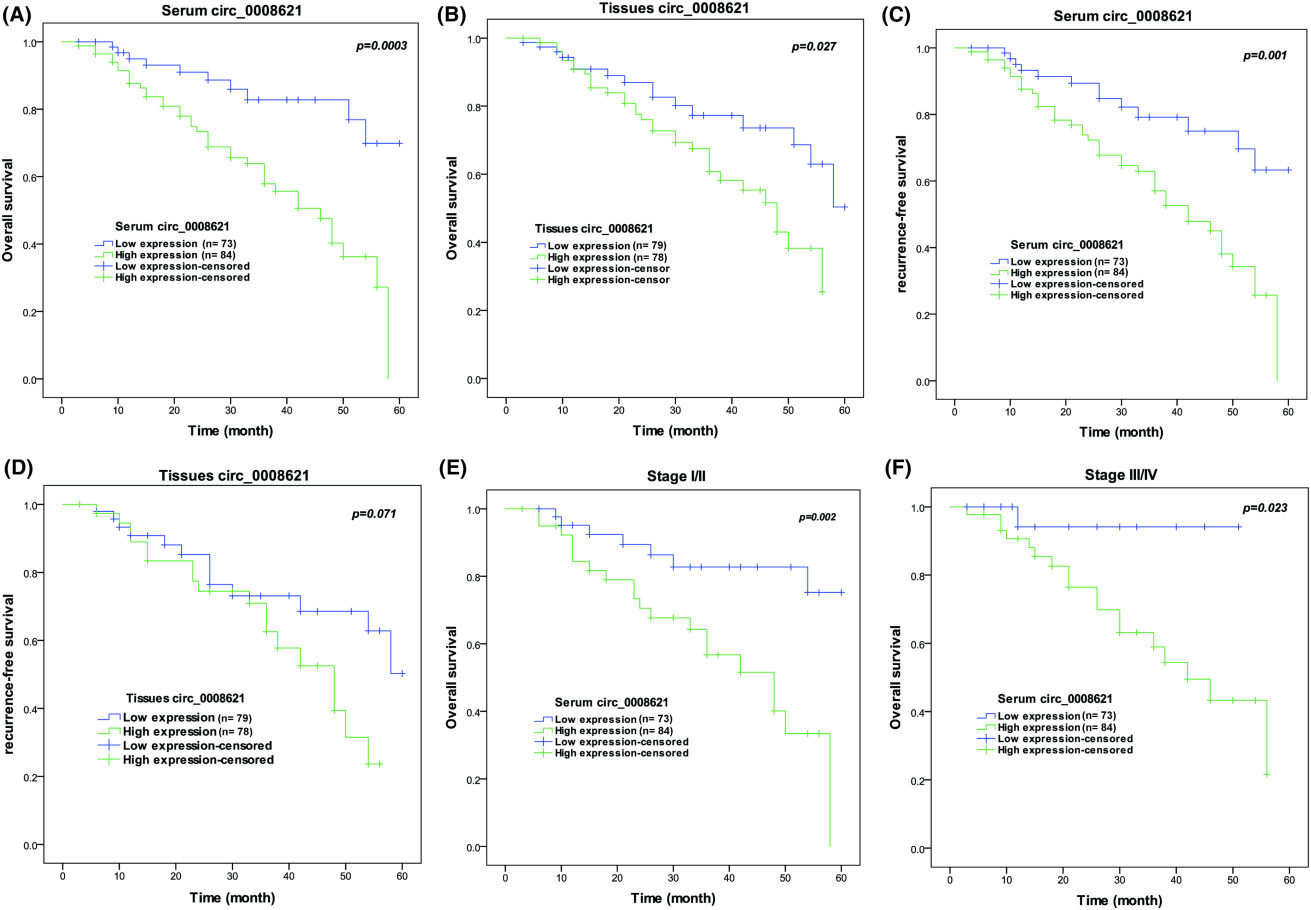
Hsa_circ_0008621 predicted poor prognosis in colorectal cancer (CRC). (A) Overall survival of CRC patients dependent on expression of serum hsa_circ_0008621 (*p* = 0.0003 by log rank test). (B) Recurrence‐free survival of CRC patients dependent on expression of serum hsa_circ_0008621 (*p* = 0.027 by log rank test). (C) Overall survival of CRC patients dependent on expression of tissue hsa_circ_0008621 (*p* = 0.001 by log rank test). (D) Recurrence‐free survival of CRC patients dependent on expression of tissue hsa_circ_0008621 (*p* = 0.071 by log rank test). (E) Overall survival of CRC patients at stage I/II dependent on expression of serum hsa_circ_0008621 (*p* = 0.002 by log rank test). (F) Overall survival of CRC patients at stage III/IV dependent on expression of serum hsa_circ_0008621 (*p* = 0.023 by log rank test).

The difference in expression of hsa_circ_0008621 in CRC tissues and serum prompted us to test whether hsa_circ_0008621 expression has the potential to serve as a prognostic biomarker for CRC patients. The level of hsa_circ_0008621 expression was categorized into low and high, respectively, based on the mean value of hsa_circ_0008621 in CRC tissues and serum. Subsequently, high hsa_circ_0008621 expression in serum was significantly associated with tumor size, N stage, M stage, and TNM stage; high hsa_circ_0008621 expression in CRC tissues was significantly associated with tumor size, M stage, and TNM stage (all *p*‐values < 0.05, Table 1). Multivariate analysis identified high expression of serum hsa_circ_0008621 as a poor prognosticator for CRC overall survival (HR: 7.334, 95% confidence interval (CI): 1.940–13.168, *p* < 0.0001), as shown in Table 2. Overall survival was significantly worse in the high serum hsa_circ_0008621 group as depicted by Kaplan–Meier curve (Figure 2A). The Kaplan–Meier curve showed high tissue hsa_circ_0008621 predicted a shorter overall survival (*p* = 0.027, Figure 2B), Multivariate analysis hasn't identified high tissue hsa_circ_0008621 as a prognosticator for CRC overall survival (Table 2). Additionally, there was a statistical significance for higher serum hsa_circ_0008621 (HR: 5.055, 95% CI: 1.940–13.168, *p* = 0.001) levels in the shorter DFS of CRC patients (Table 2). Kaplan–Meier curve supported the potential of high serum hsa_circ_0008621 expression in predicting the DFS (*p* = 0.001, Figure 2C). However, high tissue hsa_circ_0008621 can't reflect the DFS significantly (Figure 2D). Next, to determine whether the effect of serum hsa_circ_0008621 on patient overall survival was affected by the stage of tumor, the prognostic potential of serum hsa_circ_0008621 was separately evaluated in early‐stage (I/II) and advanced‐stage (III/IV) CRC patients. Interestingly, high serum hsa_circ_0008621 expression was significantly associated with poor overall survival in CRC patients at early stages (Figure 2E; log‐rank *p* = 0.002) and advanced stages (Figure 2F; log‐rank *p* = 0.023).

**TABLE 2:** Results of multivariate analysis for patients' prognosis.

Characteristics	Overall survival			Disease‐free survival		
	HR	95% CI	*p* value	HR	95% CI	*p* value
Serum hsa_circ_0008621 (High vs. Low)	7.334	2.612–20.591	<0.0001	5.055	1.940–13.168	0.001
Tissues hsa_circ_0008621 (High vs. Low)	2.234	0.914–5.464	0.078	1.981	0.844–4.648	0.116
Age (> 65 vs. ≤65)	1.201	0.648–2.225	0.560	1.017	0.567–1.824	0.9545
Sex (Male vs. Female)	1.117	0.615–2.026	0.717	1.144	0.655–1.998	0.636
Size (>Median vs. ≤Median)	1.217	0.647–2.291	0.543	1.196	0.666–2.147	0.550
T stage (T3/T4 vs. T1/T2)	1.401	0.717–2.738	0.325	1.364	0.733–2.541	0.328
N stage (N1–N3 vs. N0)	1.154	0.619–2.1525	0.652	1.123	0.625–2.019	0.698
M stage (M1 vs. M0)	3.313	1.192–9.203	0.022	1.862	0.753–4.602	0.178
TNM (III/IV vs. I/II)	1.611	0.802–3.233	0.180	1.323	0.668–2.624	0.422
CEA (>5 vs. ≤5)	1.529	0.808–2.896	0.192	1.713	0.940–3.122	0.079

### 3.3 Hsa_circ_0008621 knockdown inhibited proliferation, migration, and invasiveness of CRC cells

**FIGURE 3:**
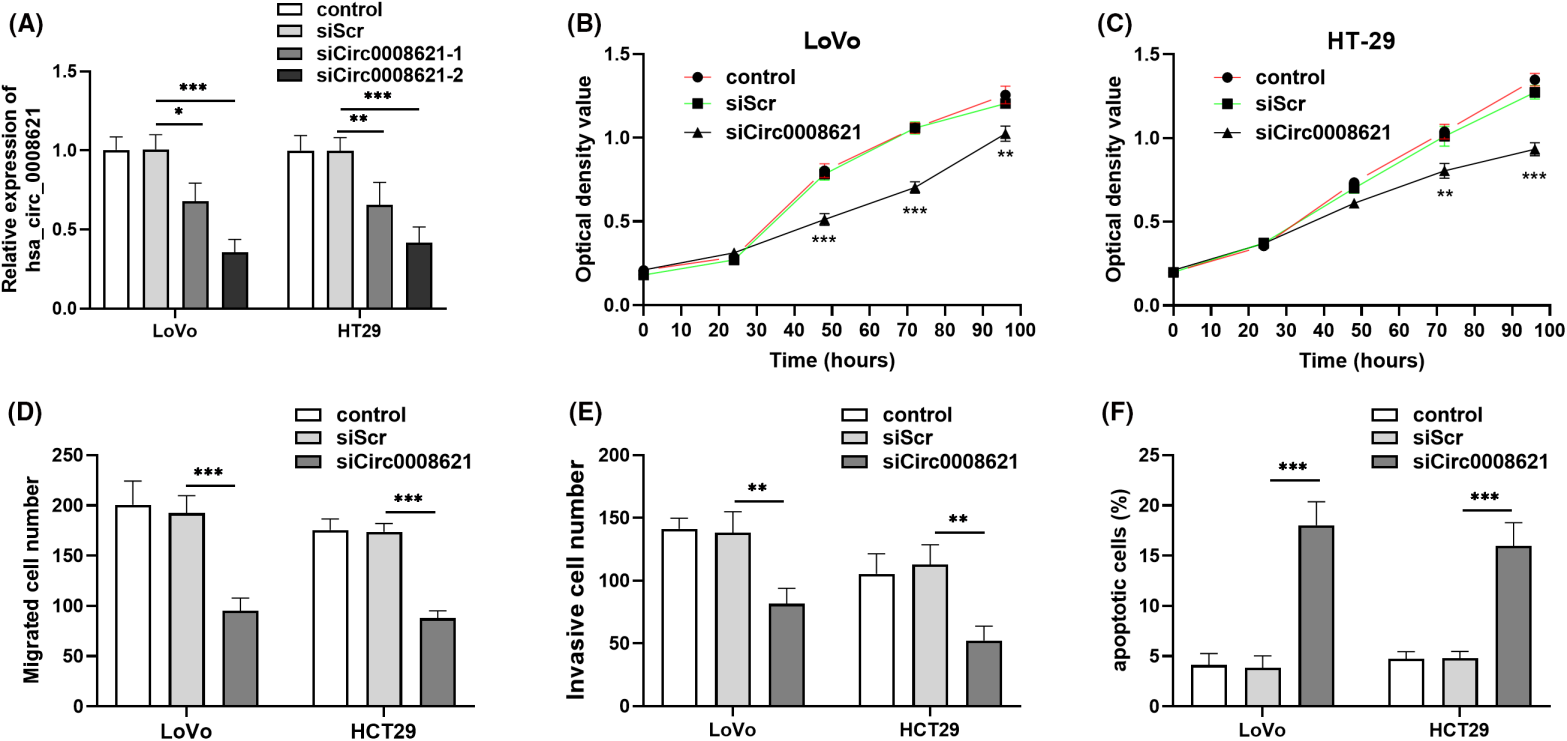
Hsa_circ_0008621 knockdown inhibited cell proliferation, migration, and invasiveness, but induced apoptosis. (A) Knockdown efficiency was measured by qRT‐PCR in LoVo and HT‐29 cells. The proliferation of LoVo (B) and HT‐29 (C) cell lines was detected at different time points using CCK‐8. Transwell assay was used to assess cell migration (D) and invasion (E). (F) Flow cytometry was performed to detect cell apoptosis. ***p < 0.001, **p < 0.01, *p < 0.05.

To determine the role of hsa_circ_0008621 in CRC cell function, we employed two hsa_circ_0008621‐specific siRNAs. As shown in Figure 3A, siCirc0008621‐2 can knockdown the hsa_circ_0008621 more than siCirc0008621‐1, thus for subsequent use, defined as siCirc0008621. Compared with negative control, growth of hsa_circ_0008621‐knockdown CRC cells was significantly reduced, analyzed by CCK‐8 assays (Figure 3B,C). Moreover, hsa_circ_0008621 knockdown led to reduced migration (Figure 3D) and invasion (Figure 3E) in LoVo and HT‐29 cells. More apoptotic LoVo and HT‐29 cells were found upon hsa_circ_0008621 knockdown (Figure 3F) detected by flow cytometric analysis.

### 3.4 Hsa_circ_0008621 knockdown attenuated glycolysis in CRC cells

**FIGURE 4:**
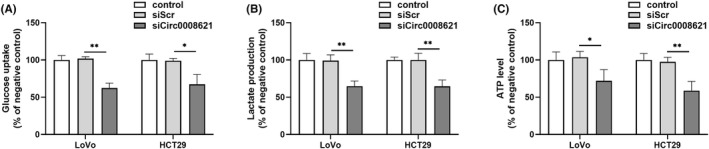
Hsa_circ_0008621 affected glycolysis in colorectal cancer cells. Glucose uptake (A), lactate production (B), and ATP production (C) measurements in LoVo and HT‐29 cells upon hsa_circ_0008621 knockdown were detected using corresponding commercialized kits. **p < 0.01, *p < 0.05.

In order to confirm the effect of hsa_circ_0008621 on glycolysis, we studied the effect of hsa_circ_0008621 knockdown on glucose uptake, lactate release, and ATP production hsa_circ_0008621 knockdown resulted in a decrease in glucose uptake (Figure 4A) and release of lactate (Figure 4B). Furthermore, a reduction in intracellular ATP levels was observed upon hsa_circ_0008621 knockdown (Figure 4C).

### 3.5 Hsa_circ_0008621 sponged miR‐532‐5p

**FIGURE 5:**
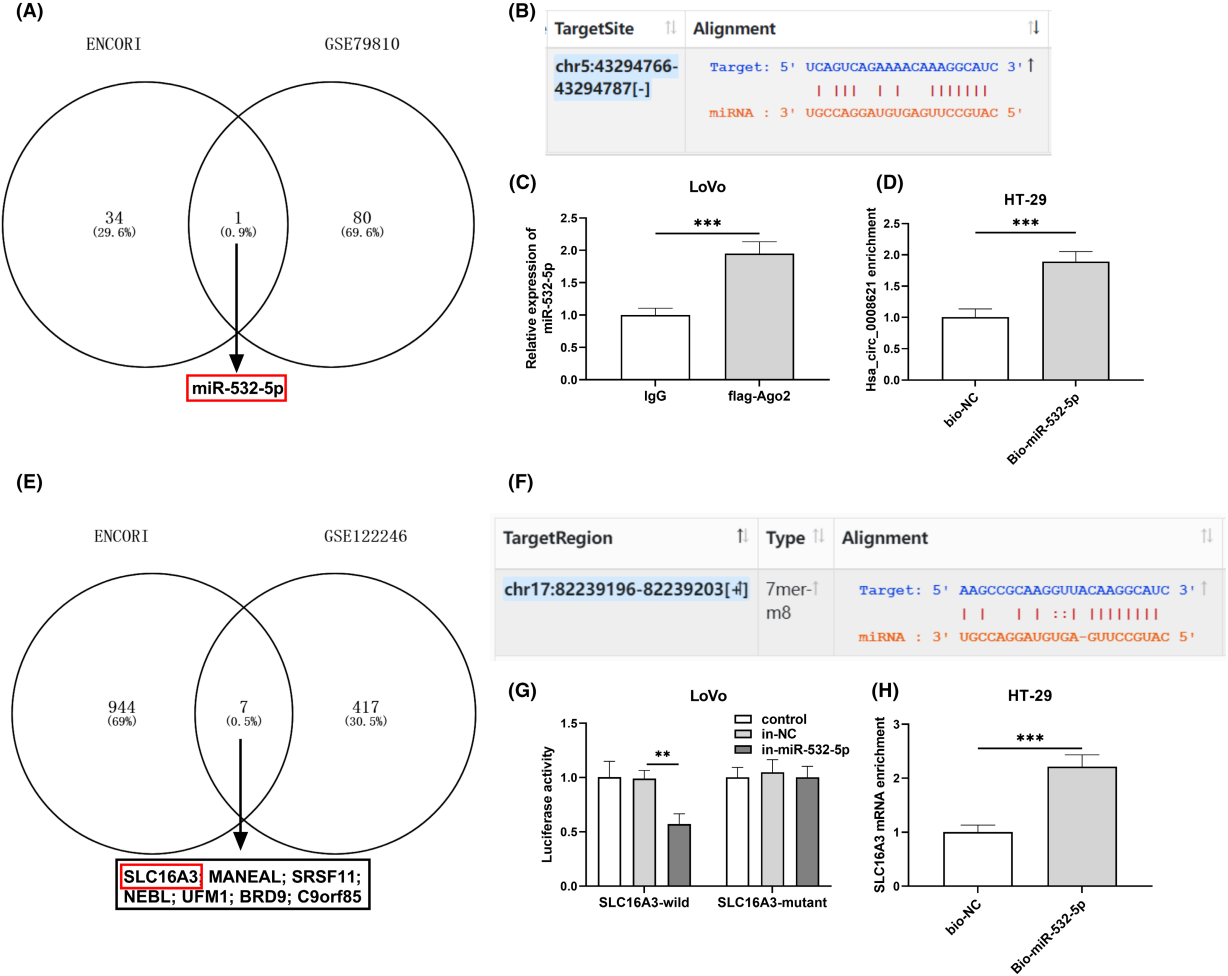
Hsa_circ_0008621 was a ceRNA for miR‐532‐5p, which targets SLC16A3, in colorectal cancer cells. (A) MiR‐532‐5p was a downstream miRNA for hsa_circ_0008621. (B) The binding sites between hsa_circ_0008621 and miR‐532‐5p were predicted on ENCORI. (C) RIP‐qPCR examined the miR‐532‐5p in hsa_circ_0008621 immunoprecipitates from LoVo cells. (D) RNA pull‐down followed by qRT‐PCR analysis examined the hsa_circ_0008621 pulled down by biotin‐labeled miR‐532‐5p probes. (E) SLC16A3 was one targeting gene for miR‐532‐5p. (F) The binding sites between SLC16A3 and miR‐532‐5p were predicted on ENCORI. (G) Dual‐luciferase reporter assays were used to validate SLC16A3 binding with miR‐532‐5p in LoVo cells. (H) RNA pull‐down followed by qRT‐PCR analysis examined SLC16A3 that was pulled down by biotin‐labeled miR‐532‐5p probes. ***p < 0.001, **p < 0.01.

For investigation of the downstream miRNA for hsa_circ_0008621, ENCORI database was retrieved, and GSE79810 dataset was screened. Finally, the shared miRNA indicated miR‐532‐5p, which was related to the CRC prognosis was a target for hsa_circ_0008621 (Figure 5A). The binding sites was shown in Figure 5B. RIP assay results confirmed that miR‐532‐5p was enriched in the AGO_2_ fraction in LoVo cells (Figure 5C). Alternatively, hsa_circ_0008621 was enriched in pull‐downs using a biotinylated miR‐532‐5p probe in HT‐29 cells (Figure 5D). Using ENCORI database and GSE122246 dataset, seven targeting genes, including SLC16A3, were screened for miR‐532‐5p (Figure 5E). The binding sites was shown in Figure 5F. In mild‐SLC16A3‐transfected LoVo cells, significant changes in luciferase activity were observed between the inhibitor control and miR‐532‐5p inhibitor, whereas no change occurred in mutant‐SLC16A3‐transfected LoVo cells (Figure 5G). Alternatively, SLC16A3 mRNA was enriched in pull‐downs using a biotinylated miR‐532‐5p probe in HT‐29 cells (Figure 5H).

## 4 DISCUSSION

In this study, we screened hsa_circ_0008621 as a differentially expressed circRNA in CRC. In our patient cohort, the upregulation of hsa_circ_0008621 was confirmed not only in tissues but also in serum. An elevated level of hsa_circ_0008621 in serum was associated with shorter OS and also shorter RFS in CRC patients. Knockdown using siRNA, qRT‐PCR, CCK‐8 assay, Transwell assay, and flow cytometry were the tools that we used to demonstrate the potential biological role of hsa_circ_0008621 in CRC cells. The results showed that hsa_circ_0008621 could facilitate cell proliferation, migration, and invasiveness of CRC cells but suppress cell apoptosis. In addition, hsa_circ_0008621 knockdown can attenuate glycolysis in CRC cells. Moreover, the interaction between hsa_circ_0008621 and miR‐532‐5p, as well as between miR‐532‐5p and SLC16A3, was investigated to preliminarily unravel the potential mechanism.

The onset of colorectal cancer is usually insidious and there is no obvious manifestation in the early stages. More than half of colorectal cancer patients have lymph node metastasis when detected.^20^ Therefore, recurrence and metastasis remain the primary causes of death in colorectal cancer patients. Therefore, finding effective prognostic markers is particularly important. CircRNA is a new type of non‐coding RNA that does not have a 5' polarity and a 3' poly A tail structure. They are connected by covalent bonds to form a closed continuous structure with high stability, offering the potential of circRNAs as biomarkers. For example, circRNA‐100876 was abnormally overexpressed in CRC, and the high expression of circRNA‐100876 worsened overall survival in CRC patients.^21^ Here, we identified the upregulation of hsa_circ_0008621 in CRC tissues and serum. This dysregulation made us further investigate the prognostic potential of hsa_circ_0008621 in CRC patients. Though the tissues hsa_circ_0008621 underperformed in predicting OS and DFS, serum hsa_circ_0008621 was identified as a potential independent prognostic factor for CRC patients.

In addition, in recent years, mounting studies have found that circRNAs are widely involved in human pathological and physiological activities, including regulating the occurrence and development of various tumors.^22^ For example, circ RNA_100367 is elevated and promotes tumor invasion and metastasis in esophageal cancer.^23^ Overexpression of Circ_CDR1as significantly promotes the proliferation and migration of liver cancer cells.^24^ In this study, we found hsa_circ_0008621 has the ability to promote the proliferation, migration, and invasiveness of the CRC cells. Its knockdown can inhibit CRC cell apoptosis. These results are in concordance with another study that reported elevated circHMGCS1‐016 promoting intrahepatic cholangiocarcinoma development both in vitro and in vivo.^18^ Moreover, we found hsa_circ_0008621 knockdown can attenuate glycolysis in CRC cells. As is known, most cancer cells rely on glycolysis, more than mitochondrial oxidative phosphorylation, to generate ATP, even in the presence of oxygen; thus, the cancerous cells exhibit increased glucose uptake and lactate production.^25^ In this study, our results revealed that hsa_circ_0008621 knockdown can decrease the glucose uptake, release of lactate and intracellular ATP levels. Overall, these results indicate that hsa_circ_0008621 knockdown can diminish cell malignant phenotype of CRC cells.

In recent years, increasing studies have shown that circRNA can act as a sponge molecule to adsorb miRNA, thus affecting the expression of miRNA and playing a biological role in cells. In this study, we predicted prognosis‐related miRNAs targeting hsa_circ_0008621 ENCORI and GEO database. MiR‐532‐5p was predicted and then verified to be downstream miRNA for hsa_circ_0008621. MiR‐532‐5p overexpression suppressed K‐Ras/Erk1/2 and PI3K/Akt signaling pathways in HCT116 cells and inhibit cell cycle progression.^26^ After the confirmation of hsa_circ_0008621 as ceRNA for miR‐532‐5p, our next focus is to search for downstream target genes for miR‐532‐5p. After bioinformatics analysis, we confirmed through Luciferase assay and pulldown analysis that miR‐532‐5p can specifically bind to SLC16A3. SLC16A3, also known as monocarboxylate transporter 4, is responsible for the extracellular transport of lactate, involved in glycolysis. SLC16A3 predicts a worse prognosis in lung cancer and may involve in immune microenvironment in lung cancer.^27^ Thus, hsa_circ_0008621 may contribute to CRC progression through miR‐532‐5p/SLC16A3.

In conclusion, serum hsa_circ_0008621 could be of potential use in predicting overall survival and recurrence‐free survival for CRC patients. Hsa_circ_0008621 knockdown could be especially helpful for the therapy of CRC patients. These findings would improve the understanding of CRC pathophysiology and open the door to the discovery of new CRC biomarkers and therapeutic targets.

## AUTHOR CONTRIBUTIONS

X.H. Zhou, L. Wu, and C.Y. Tian carried out the concepts, design, and definition of intellectual content. X.H. Zhou and L. Wu provided assistance for data acquisition, data analysis, and statistical analysis. All authors performed the experiment, and draft of the manuscript. C.Y. Tian revised the manuscript critically for important intellectual content. All authors have read and approved the content of the manuscript.

## FUNDING INFORMATION

No funds, grants, or other support was received.

## CONFLICT OF INTEREST STATEMENT

The authors declare no conflicts of interest for this article.

## ETHICS STATEMENT

Approval of the research protocol: This study was performed in line with the principles of the Declaration of Helsinki. The study was approved by the ethical committees of The Affiliated Xuzhou Municipal Hospital of Xuzhou Medical University.

Informed Consent: N/A.

Registry and the Registration No. of the study/trial: N/A.

Animal Studies: N/A.

## Supporting information


Figure S1

Table S1
