# Supplementary figures and images for: Overexpression of circular RNA hsa_circ_0008621 facilitates colorectal cancer progression and predicts poor prognosis
Source: Ann Gastroenterol Surg. 2024 Mar 22;8(4):639–49. doi: 10.1002/ags3.12793 (PMC11216790; doi:10.1002/ags3.12793)

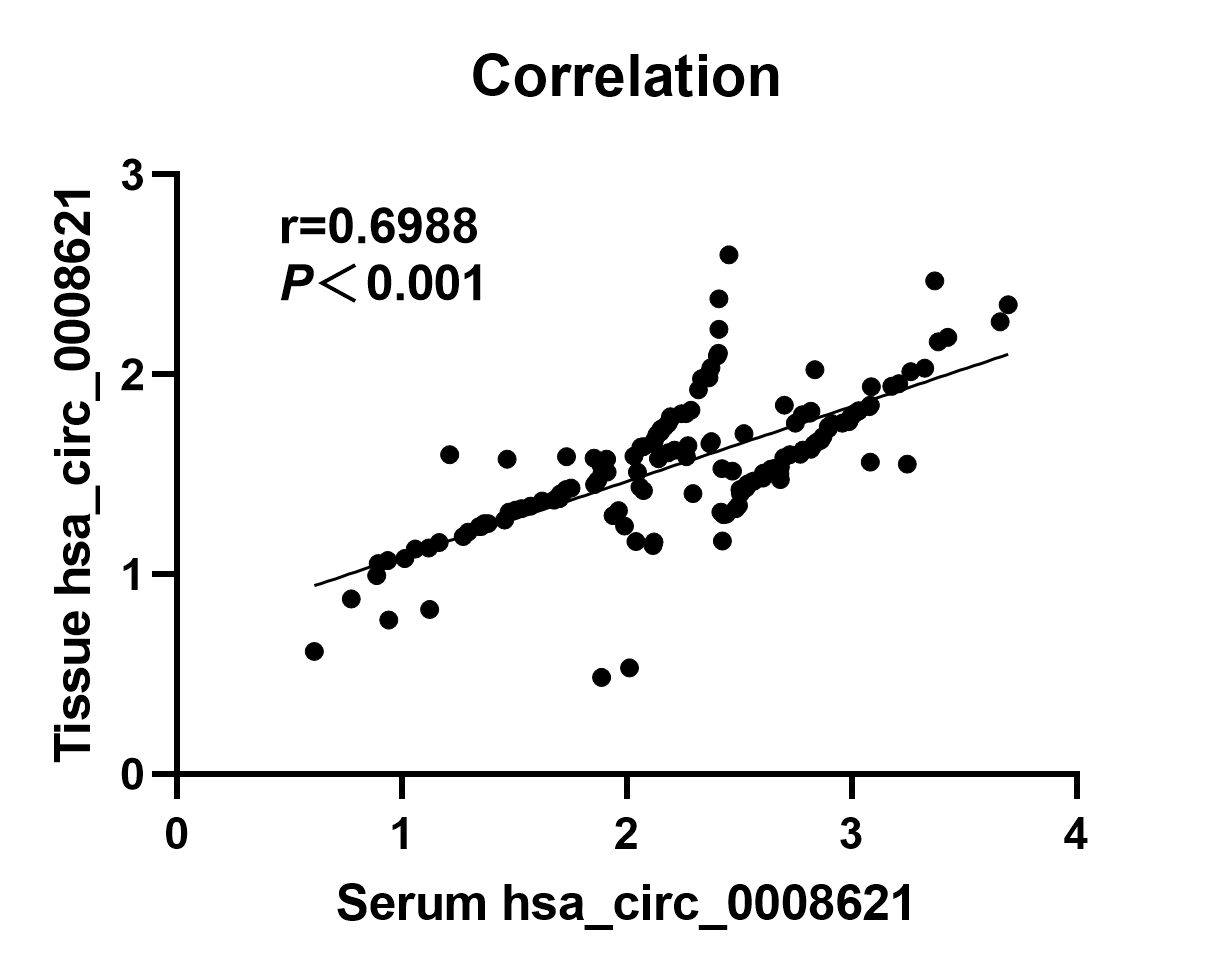

Supplement: Supplementary file 1 — Figure S1 [file AGS3-8-639-s001.tif]
